# Effects of prenatal auditory stimulation on physiological stress and neurotransmitter levels in developing embryos and day-old ducklings

**DOI:** 10.5713/ab.25.0243

**Published:** 2025-09-30

**Authors:** Yashini Subramaniam, Suriya Kumari Ramiah, Intan Shameha Abdul Razak, Aimi Nabilah Hussein, Ahmad Hanafi Sulong, Zulkifli Idrus

**Affiliations:** 1 Institute of Tropical Agriculture and Food Security, Universiti Putra Malaysia, Serdang, Malaysia; 2 School of Veterinary Medicine, International Medical University, Kuala Lumpur, Malaysia; 3 Department of Veterinary Pre-Clinical Sciences, Faculty of Veterinary Medicine, Universiti Putra Malaysia, Serdang, Malaysia; 4 Department of Animal Science, Faculty of Agriculture, Universiti Putra Malaysia, Serdang, Malaysia; 5 School of Animal Science, Faculty of Bioresources and Food Industry, Universiti Sultan Zainal Abidin, Besut, Malaysia

**Keywords:** Ducks, Embryos, Prenatal Auditory Stimulation, Stress

## Abstract

**Objective:**

The objective of the study was to determine the effects of prenatal auditory stimulation (PAS) on serum concentrations of heat shock protein 70 (HSP70), ceruloplasmin (CPN), alpha-1-acid-glycoprotein (AGP), corticosterone (CORT), ovotransferrin (OVT), and dopamine (DA) in duck embryos and hatchlings.

**Methods:**

Fertilized Khaki Campbell duck eggs were subjected to the following auditory stimulation treatments: 1) no supplementary sound treatment other than the background sound of the incubator's compressors at 40 dB (CONTROL), 2) exposure to pre-recorded traffic noise at 90 dB (NOISE), and 3) exposure to Mozart's Sonata for Two Pianos in D Major, K 488 at 90 dB (MUSIC). The NOISE and MUSIC treatments were lasted for 20 min per hours over a 24 h (a total of 8 h/d), starting from embryonic days (ED) 13 to hatching. PAS did not influence hatchability rate, body weights at ED 21 and post-hatch day (PH) 1.

**Results:**

The MUSIC and NOISE treatments elevated HSP70 at ED21 compared to the Control, while opposite results were observed at post-hatch day 1 (PH1). The AGP, CPN and CORT were not significantly (p>0.05) affected by PAS at ED21. However, at PH1, the Control ducklings showed significantly higher OVT and AGP. The NOISE and MUSIC treatments reduced DA activity in ED21 and PH1, respectively.

**Conclusion:**

PAS induces a physiological stress response in embryos without affecting hatchability or the weights of hatchling. Exposure to MUSIC and NOISE modulates stress-related physiological markers during incubation and hatching, hence enhancing stress resilience in day-old ducklings.

## INTRODUCTION

Prenatal stress in poultry could originate from various environmental factors such as temperature fluctuations, inadequate nutrition, and exposure to toxins, as well as from the physiological state of the hen, including her health, stress levels, and overall well-being [1]. Stress during embryonic development plays a significant role in shaping the physiological and psychological development of the chick, potentially leading to long-term effects on growth rates, immune function, behaviour and stress responsiveness [2].

While prenatal stress in poultry is generally associated with adverse outcomes, there is some evidence suggesting that mild or controlled stress exposure during embryonic development can have potential benefits [3]. Recently, Ahmad-Hanafi et al [4] showed that prenatal auditory stimulation (PAS) enhanced the ability of broiler chickens to cope with the stress associated with feed deprivation at market age. Earlier studies found that prenatal sound stimulation with sitar music and species-specific calls improves chicks’ hippocampus development and learning ability at 12 h post-hatch [5]. Donofre et al [6] found that loud noise exposure during embryonic development improved hatchability rates and chick quality.

Given its significant implications for poultry health, productivity, and welfare, elucidating the consequences of PAS on physiological stress response in poultry is crucial. In domestic chickens, the hearing system began to develop as early as day 10 of incubation [7], and the chicken embryo began to respond on day 16 of incubation to external sounds that were lower than 90 dB [8]. Hanafi et al [9] noted that developing chicken embryos reacted to noise and music exposure by altering serum levels of corticosterone (CORT), heat shock protein (HSP) 70, and acute phase proteins (APPs), which are crucial for managing stress due to auditory stimulation.

Despite the growing demand for duck meat, recent reviews of Pekin ducks in commercial systems have pointed out a significant gap in research on their well-being, especially when compared to other poultry species [10]. Thus, the objective of this study was to examine the effects of PAS on physiological stress response in duck embryos and day-old ducklings. By understanding the effects of prenatal stress, more effective welfare practices can be designed to reduce stress and its negative impacts, ultimately improving the overall well-being of meat ducks. Serum levels of CORT, APPs (ceruloplasmin [CPN], alpha-1-acid glycoprotein [AGP], ovotransferrin [OVT]), HSP70, and dopamine (DA) were used as biomarkers of physiological stress in the present study.

## MATERIALS AND METHODS

### Experimental incubators

Six Brinsea Ovo-Easy Incubators (Brinsea Products), each with a holding capacity of 144 eggs, were used. These incubators were outfitted with both electronic temperature and humidity controls, as well as three trays on tilted platforms that allowed the eggs to be turned as preset. Each incubator had a preset incubation temperature of 37.8°C with a precision of 0.5°C. The average relative humidity should be at 55% on embryonic day (ED) 1 and range between 60% to 65% on ED 25 until–hatching. Every two hours, eggs were automatically rotated from ED 1 to ED 25. On ED 26, eggs were gently moved onto hatching trays and not rotated for the rest of the incubation period. All incubators were located in the same ventilated room with a controlled environmental temperature of 24°C to 25°C (accuracy of 2°C). The incubators were installed with double-walled, soundproof medium-density fiberboard insulators in order to shield sound treatment from interference. Sound pressure levels (SPLs) were measured inside and outside the operating incubators using a sound meter. The SPL outside the operating incubators dropped below 50 dB, while inside, the operating incubator was maintained at 90 dB.

### Auditory stimulation protocols

The background noise was assessed at the centre of the incubation chambers using a digital sound level meter (TECPEL DSL-330). Sound treatments were delivered inside the chambers through a 10 W wireless speaker (Tronsmart Element Groove) with a continuous 24-hour playback capability. The speaker, positioned inside the incubation chamber, played sounds stored in MP3 format at a 44.1 kHz sampling frequency on an SD card. The intensity of the emitted sounds was calibrated at the start of the exposure using the sound level meter placed at the chamber’s centre. The MP3 recordings of music and noise signals were analysed using MATLAB software (ver. 2020B) running on a computer equipped with an Intel Core i7 CPU (2.6 GHz) and 16 GB RAM. The right-channel signal spectrums were examined using the fast Fourier transform (FFT) and short-time Fourier transform (STFT) methods, utilising FFT and spectrogram functions from the signal processing toolbox. To generate the spectrogram, a Hamming window of 1,000 lengths, with a 50% overlap and 256 FFT points, was applied. The spectrogram characteristics of the music and noise signals were as described by Hanafi et al [9]. The total signal energy was determined by summing the energy at individual frequencies, following the approach outlined by McLoughlin [11].


(1) $$E=\frac{1}{N}\sum_{k=1}^{N}{\left|X\left(f_{k}\right)\right|}^{2}$$

where *X*(*f_k_*), *f_k_*, and *N* is the spectral amplitude density of discrete frequency and the number of discrete frequency elements, respectively. Hanafi et al [9] indicated that the total energy of the noise, 0.2898 Joules, was higher than that of the music, 0.1044 Joules.

### Experimental groups

The hatching duck eggs were subjected to one of the following treatments [9]:

1) Control: The eggs were incubated and received no additional sound stimuli except the compressor’s (40 dB) sound, which was unavoidable throughout the incubation period.
2) Music stimulation: The eggs were incubated under conditions similar to the Control. Starting from ED15 to ED25, the eggs were exposed to Mozart’s Sonata for Two Pianos in D Major, K 488 (90 dB) at the frequency range of 100–6,300 Hz for 20 minutes per hour over 24 hours (a total of 8 hours per day). The experimental treatment was referred to as the “MUSIC” group. The last cycle of music stimulation was approximately at 9:00.
3) Noise stimulation: The eggs were incubated under conditions similar to those of controls and music groups. Starting from ED15 to ED25, the eggs were exposed to pre-recorded vehicle horn sound (90 dB) at the frequency range of 100–6,300 Hz for 20 minutes/hour over 24 hours (a total of 8 h/day). The experimental treatment was referred to as the “NOISE” group. The last cycle of music stimulation was approximately at 9:00.

The PAS treatments followed the protocol described by Hanafi et al [9] for chicken embryos, with adjustments to the timing of exposure to account for species-specific differences in incubation periods. In the present study, similar treatments were applied to duck embryos based on the assumption that auditory stimulation elicits broadly comparable developmental responses across avian species.

### Pre-hatching responses

A total of 360 Khaki Campbell duck-hatching eggs (60–75 g) were purchased from a local commercial hatchery. The eggs were fumigated and incubated in six double-insulated incubators (Brinsea Ovo-Easy 190 Advance Series) with a capacity of 100 eggs/incubator. The incubator used a forced draft of air for ventilation, aiming to maintain uniform temperature and humidity levels throughout the chamber. At ED 14, all eggs were candled, and 24 infertile eggs or eggs with dead embryos were removed from the incubators. The remaining 336 eggs were equally allotted to 6 similar incubators (56 eggs per incubator). Commencing from ED 15, eggs from the NOISE and MUSIC groups were exposed to background noise and music at 90 dB emitted by the portable speaker placed in the assigned incubators. Maturation of the embryo’s auditory system occurred at ED 15 [12]. Auditory stimuli were emitted at 90 dB for 20-minute intervals every hour from ED 15 to ED 28 at recorded SPLs. Blood sampling was carried out on 15 individual embryos from each treatment group on ED 21. The embryos were removed from the eggs, weighed and blood was collected directly into 10×75 mm centrifuge tubes from the vitelline artery and vein. The samples were then centrifuged at 3,000×g for 10 min at room temperature to separate the serum. Serum samples were kept at −80°C until analysed. Serum samples were used to determine CPN, AGP, CORT, OVT, HSP70 and DA.

### Hatching responses

The remaining 336 fertile eggs were allowed to hatch in the incubator. During hatching, the number of ducklings hatched was recorded every hour. The hatching windows were between day 27th and day 28th of incubation. The percentage of hatchability was calculated according to the formula given below:


(2) $$\text{Hatchability }(\%)=\frac{\text{Number of ducklings hatched}}{\text{Number of fertile eggs loaded in incubator}}\times 100$$

After feather drying (approximately 2 hours after hatching), the ducklings were individually weighed. Fifteen ducklings per treatment group (a total of 45 ducklings) were randomly selected, and their blood samples were collected by decapitation upon hatching. The blood samples were then centrifuged at 3,000×g for 10 min at room temperature to separate the serum. Serum samples were kept at −80°C until analysed. Serum samples were used to determine CPN, AGP, CORT, OVT, HSP70, and DA.

### Determination of circulating corticosterone, acute phase proteins, heat shock protein 70 and dopamine

#### Corticosterone assay

According to manufacturer recommendations, the CORT level was determined using commercially available high-sensitivity EIA kits (AC-15F1, IDS). The cross-reactivity of the CORT antiserum was less than 6.7% and 7.8%, respectively, and the detection limit was 27 ng/mL.

#### Acute phase proteins assay

The AGP level was determined using a commercial ELISA kit specific to ducks (Cat. No. 201-28-0178; Shanghai SunRed Biological Technology). Meanwhile, the level of CPN was determined, as previously explained [13]. About 20.375 g of sodium acetate trihydrate was dissolved in 250 mL of distilled water and adjusted to pH 6.2 using glacial acetic acid. Subsequently, 0.615 g of 1,4-phenylenediamine dihydrochloride (Sigma P1519; Sigma Aldrich) was added to the prepared buffer and kept in the dark for at least 45 min. Then, 100 mL of the described buffer was mixed with 10 mL of samples or standards and added to each microplate well, gently shaken and kept in the dark for 20 min. After 20 min, the plate was read using a microplate reader at 550 nm (Multiskan FC Microplate Photometer). A serial dilution of standards was prepared using a known CPN concentration against purified CPN (Sigma-Aldrich). In order to reach concentrations of 12.75 mg/mL of CPN, 20 mL of pig serum was added to 60 mL of saline buffer. Serial dilutions were prepared accordingly to reach various concentrations of 6.375, 3.1875, 1.59375, 0.79608, 0.39804, 0.199, and 0.099 mg/mL of CPN. The OVT level of ducklings’ blood serum was determined using a commercial ELISA kit (Cat. No. 201-16-3562; Shanghai SunRed Biological Technology).

#### Heat shock protein 70 assay

HSP70 determination was performed using a commercial ELISA kit specific to ducks (Cat. No. 201-28-0905; Shanghai SunRed Biological Technology) according to manufacturer recommendations. All samples were run in the same assay to prevent inter-assay variability.

#### Dopamine assay

DA determination was performed using a commercial ELISA kit specific to duck (BA E-5300R; Labor Diagnostika Nord) according to manufacturer recommendations.

### Statistical analysis

All statistical analyses were performed with the aid of Statistical Analysis System (SAS Institute) software, using 1-way ANOVA. When the analysis of variance indicated the presence of significant differences between the treatments, means were separated using the Duncan multiple range test. The hatchability data were subjected to a chi-square test. Significant differences among means were determined based on a probability of p<0.05.

## RESULTS

**Figure 1:**
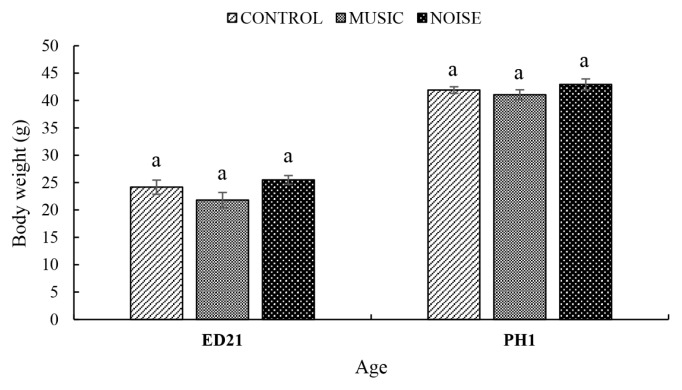
The effect of prenatal auditory stimulation on the body weights of duck embryos and day-old ducklings. ^a^ Means are not significantly different (p>0.05).

The Control (86.8%) MUSIC (77.6%) and NOISE (83.6%) groups had a similar (p = 0.38) percentage of hatchability. The body weights of duck embryos on ED 21 (Control, 24.17±1.3 g; MUSIC, 21.81±1.4 g; NOISE, 25.49±0.8 g) and day-old ducklings on post-hatch day 1 (PH1; Control, 41.91±0.6 g; MUSIC, 41.05±0.9 g; NOISE, 42.94±1.0 g) were not significantly (p>0.05) influenced by the PAS (Figure 1).

**Figure 2:**
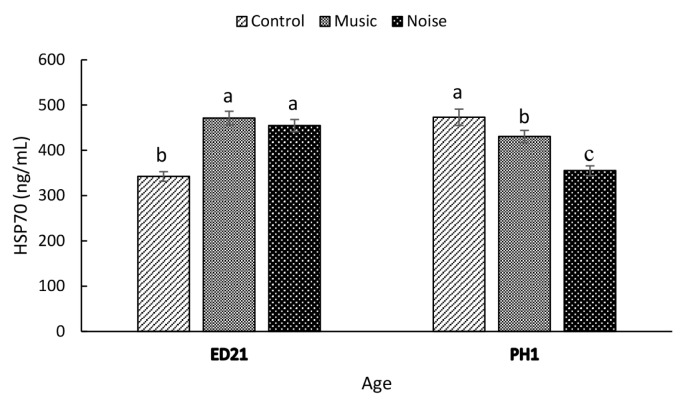
The effect of prenatal auditory stimulation on serum levels of heat shock protein (HSP) 70 in duck embryos and day-old ducklings. ^a–c^ Means with different letters significantly differ at p≤0.05.

The MUSIC and NOISE embryos had significantly higher (p = 0.0001) HSP70 expression **(**Figure 2**)** than their Control counterparts on ED21 (Control, 342.50±10.7 ng/mL; MUSIC, 471.25±15.1 ng/mL; NOISE, 454.85±13.34 ng/mL). On PH1, the Control ducklings (473.13±17.9 ng/mL) had the highest (p = 0.0001) HSP70 expression, followed by the MUSIC (430.63±13.5 ng/mL) and NOISE (355.21±10.73 ng/mL) groups.

**Figure 3:**
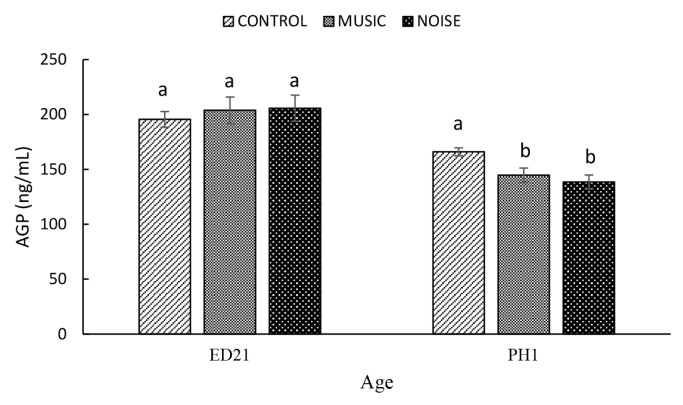
The effect of prenatal auditory stimulation on serum levels of α 1-acid glycoprotein (AGP) in duck embryos and day-old ducklings. ^a,b^ Means with different letters significantly differ at p≤0.05.

**Figure 4:**
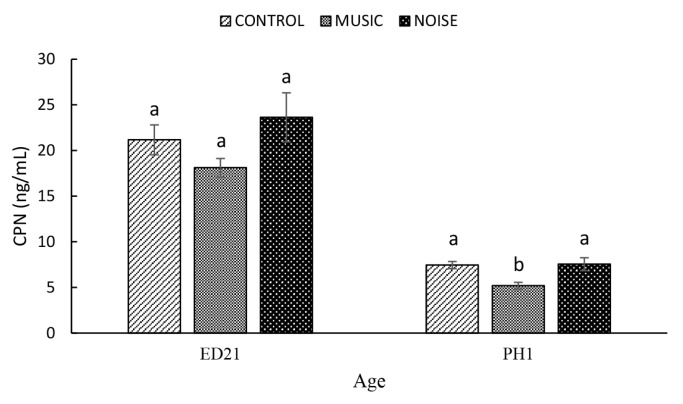
The effect of auditory stimulation on serum levels of ceruloplasmin (CPN) in duck embryos and day-old ducklings. ^a,b^ Means with different letters significantly differ at p≤0.05.

The PAS did not significantly affect (p = 0.7895) AGP (Control, 195.57±7.2 ng/mL; MUSIC, 203.86±12.1 ng/mL; NOISE, 205.72±11.9 ng/mL) **(**Figure 3**)** and CPN (CPN: Control. 21.17±1.6 ng/mL; MUSIC, 18.11±1.0 ng/mL; NOISE, 23.64±2.7 ng/mL) **(**Figure 4**)** on ED21 However, the AGP was significantly lower (p = 0.0129) in the MUSIC (144.75±6.5 ng/mL) and NOISE (138.50±6.4 ng/mL) ducklings at PH1 when compared with that of their Control (165.97±3.6 ng/mL) counterparts. On the other hand, the CPN in the MUSIC embryos (5.19±0.4 ng/mL) was significantly lower when compared to the Control (7.45±0.4 ng/mL) and NOISE (7.55±0.7 ng/mL) groups at PH1 (p = 0.1248). The Control and NOISE ducklings showed a similar (p = 0.0026) CPN on PH1.

**Figure 5:**
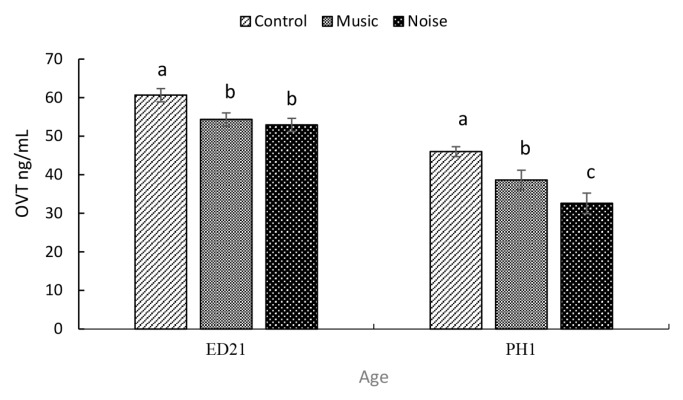
The effect of auditory stimulation on serum levels of ovotransferrin (OVT) in duck embryos and day-old ducklings. ^a–c^ Means with different letters significantly differ at p≤0.05.

On ED21, the NOISE and MUSIC embryo groups had significantly lower (p = 0.0025) OVT (Figure 5) than those of the Control (Control, 60.64±1.5 ng/mL; MUSIC, 54.33±1.1 ng/mL; NOISE, 52.91±ng/mL) group. The NOISE (34.55±2.7 ng/mL) and MUSIC (47.70±2.5 ng/mL) ducklings showed a significant (p = 0.0001) decline in OVT when compared to their Control counterparts (45.99±1.3 ng/mL) on PH1.

**Figure 6:**
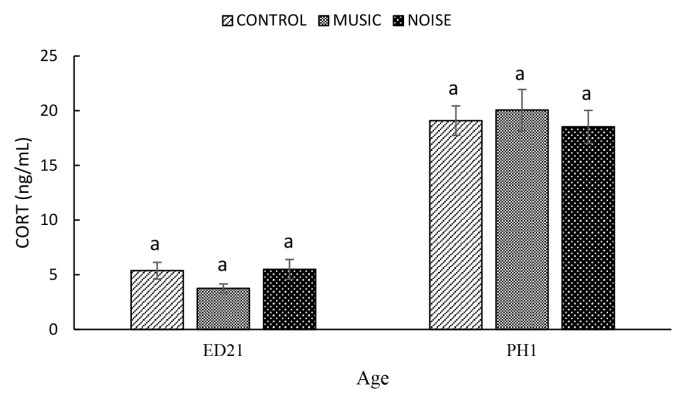
The effect of prenatal auditory stimulation on serum levels of corticosterone (CORT) in duck embryos and day-old ducklings. ^a^ Means with different letters significantly differ at p≤0.05.

The PAS had a negligible (p = 0.275) influence on CORT at ED21 (Control, 5.38±0.7 ng/mL; MUSIC, 3.76±0.4 ng/mL; NOISE, 5.50±0.9 ng/mL) and PH1 (Control, 19.09±1.4 ng/mL; MUSIC, 20.06±1.9 ng/mL; NOISE, 18.54±1.5 ng/mL) (Figure 6).

Exposing embryos to MUSIC (7.67±0.6 pg/mL) resulted in significantly lower DA compared to the Control (10.17±0.7 pg/mL) and NOISE (9.22±0.7 pg/mL) groups on ED21 (p = 0.0703) (Figure 6). On the contrary, at PH1, DA was significantly lower in NOISE (11.12±0.4 pg/mL) embryos compared with that of the Control (12.86±10.3 pg/mL) and MUSIC (12.63±8.2 pg/mL) embryos (p=0.0531).

## DISCUSSION

**Figure 7:**
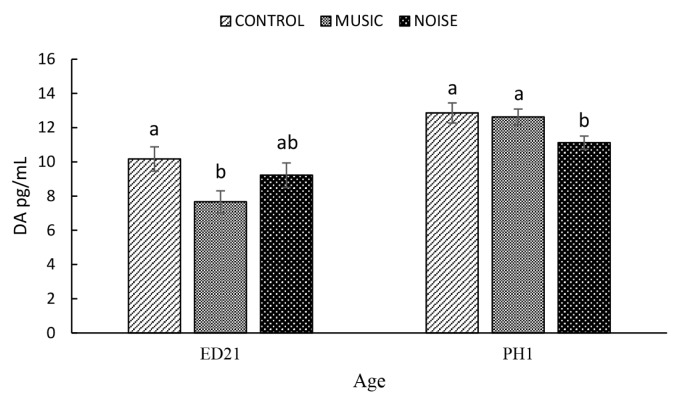
The effect of prenatal auditory stimulation on serum levels of dopamine (DA) in duck embryos and day-old ducklings. ^a,b^ Means with different letters significantly differ at p≤0.05.

When discussing the results of this study, it is essential to note that although MUSIC and NOISE were held constant at 90 dB, the total energy of the two stimuli differed [9]. In this context, NOISE had higher total energy because it is spectrally broad and temporally continuous. In contrast, MUSIC exhibited more structured intensity peaks, reaching higher energy levels only during specific instrumental overlaps. In their review, Ising and Kruppa [14] suggested that SPL alone does not adequately capture the biological impact of acoustic stimuli, as stress responses are also influenced by spectral composition, temporal characteristics, and exposure duration, even when SPL remains constant. Noise is defined as an undesired sound, whether continuous or sporadic. It can be characterised by factors such as its frequency, intensity, frequency spectrum, and the pattern of sound pressure over time [15]. Noise can significantly affect the well-being of farm animals in various ways, primarily through physiological stress, behavioural changes, and physical health impacts [16]. The present findings concur with earlier work in chickens [5,6,9] that PAS did not affect the weight of embryos and hatchlings or the hatchability rate. Thus, although PAS could be stressful to the embryos, the treatment had no adverse effect on the liveability and weight of the embryos and day-old ducklings (Figure 7).

Circulating levels of CORT can be considered a reliable physiological indicator of stress in developing poultry embryos. Stresses associated with heat [17], lighting [18], and electromagnetic fields [19] have been reported to elevate blood CORT in developing chicken embryos. Recently, Hanafi et al [9] concluded that prenatal exposure to music and noise at 90 dB, as measured by CORT, was stressful to the embryos at certain developing stages. In the present study, neither NOISE nor MUSIC had any effect on CORT. It is not clear why PAS had a negligible impact on CORT at ED 21in this study. The lack of changes in CORT response following MUSIC and NOISE exposure, while unexpected, does not preclude the possibility of prior elevation in CORT. This is because of a marked elevation in HSP70 following exposure of developing duck embryos to MUSIC and NOISE on ED21, suggesting that the PAS had induced an adrenocortical response. Zulkifli et al [13] reported that daily CORT injections for 4 and 7 days significantly increased HSP70 expression in the brain. The authors concluded that the elevated protein density in the brain is directly linked to circulating CORT levels. Studies involving rats have indicated that exposure to loud noise, typically between 80 and 110 decibels, triggered a significant upregulation of the HSP70 gene within the cochlea [20]. This increase in gene expression may act as a compensatory mechanism, aiming to counterbalance the adverse effects of mechanical stress experienced by the auditory sensory organ.

Acute-phase proteins, primarily produced in the liver, serve as important indicators of inflammation and are crucial mediators of the innate immune response in poultry. They aid host defence by inhibiting microbial growth, activating the complement system, neutralising enzymes, modulating immunity, binding cellular debris, and scavenging free radicals [21]. Earlier studies have demonstrated that serum levels of APPs increase in chickens following immune challenges, and that these rises often correlate with the antibody response. For example, Janmohammadi et al [22] observed an increase in serum levels of APPs in chickens after vaccination, and these rises are positively associated with the heterophil-to-lymphocyte ratio and antibody production.

Stress-induced elevations in APPs could be associated with the restoration of homeostasis and normal adaptation [23]. Recent studies in poultry showed that overcrowding [24], feed deprivation [25], high ambient temperature [26] and CORT administration [13] elevated serum levels of APPs. Acute-phase proteins may potentially have physiological implications for developing embryos due to their nonspecific antibacterial activities and relationship with homeostasis restoration [23]. Hanafi et al [17] reported that AGP rose from ED 14 to ED 18 and was followed by a sharp decline in the newly hatched chicks. Chick embryos exhibited elevated levels of AGP and CPN in response to MUSIC and NOISE exposure at ED15 [9]. In the present study, at ED21, the AGP and CPN levels in duck embryos were similar among the treatments. These discrepancies could be attributed to species differences [27]. For example, Petersen et al [28] worked with various mammalian species and concluded that the response pattern of the APPs is species-specific. Working with avian species, Tetel et al [29] reported that chickens exhibited significantly higher circulating CORT levels than ducks following adrenocorticotropic hormone administration and one hour of road transport, suggesting that ducks are better able to cope with acute stressors. Hence, the discrepancies in AGP and CPN findings between the present study and those reported by Hanafi et al [9] in chickens may reflect species-specific differences in stress resilience, with evidence suggesting that such interspecies variation in stress responses is already apparent at the embryonic stage.

It is noteworthy that the Control embryos exhibited significantly higher OVT compared to their MUSIC and NOISE counterparts on ED21. OVT, an iron-binding protein, possesses antimicrobial properties through iron sequestration and has been shown to modulate heterophil and macrophage functions in chickens [23]. While environmental stressors [24,25] and CORT treatment [13] have been linked to elevated OVT, increases can also be associated with inflammation and infection [30]. However, considering the noted negligible impact of PAS on hatchability rates and weights of embryos, it is unlikely that the observed OVT elevations in the developing embryos were a response to inflammation or infection.

To date, no studies have explored the effects of PAS on immune function in ducks or other avian species beyond APPs. Working with rodents, Kay et al [31] demonstrated that prenatal exposure to noise and light stress in rats impaired the immune function of their offspring, including reduced natural killer cell activity and diminished splenic lymphocyte proliferation. However, there may be differences in the impact of prenatal stress on immune responses between rats and birds, likely due to species-specific developmental pathways. In mammals, embryos are influenced by maternal hormones during gestation [32]. In contrast, avian embryos develop externally and may interpret sound as enrichment rather than a source of stress [33]. Additionally, the avian immune system matures primarily after hatching, which may reduce the long-term effects of prenatal stimuli. The impact of PAS on immunomodulatory function in poultry merits further investigation.

DA is a catecholamine released from peripheral systems such as the adrenal medulla via the sympathetic-medullary-adrenal axis. It is also a monoamine neurotransmitter primarily known for its roles in memory, learning, movement, and reward behaviours [34] and for supporting effective coping mechanisms and overall well-being [35]. DA has also been linked to auditory functions. Studies have identified DA synthesis enzymes and receptors in key auditory regions, including the inferior colliculus, cochlear nucleus, superior olivary complex, and auditory cortex, highlighting its involvement in hearing processes [36]. Moraes et al [37] reported that exposure to rhythmic music elevated DA activity in the forebrain of rats. In the current study, NOISE and MUSIC attenuated DA activity at ED21 and PH1, respectively. Working with rats, Wilson and Apawu [38] demonstrated that loud noise (10 kHz, 118 dB SPL for four hours) significantly reduced DA neurotransmission in the auditory brain region. The authors suggested that the noted changes in the DA system may be influenced by various mechanisms, including oxidative stress induced by harmful noise levels. In the present study, as measured by HSP70, the NOISE and MUSIC exposure was stressful to the developing embryos. However, the lack of differences in hatchability rate and body weights of hatchlings among the CONTROL, MUSIC and NOISE groups suggests that the auditory stimulation had no adverse effects. Hence, it appears that moderate-intensity PAS (90 dB), which did not impair hatchability or body weight, may also depress DA in embryos and hatchlings. These findings warrant caution, as elevated serum DA levels can have both beneficial and adverse effects, depending on the context, magnitude, and duration of the elevation. Moderate increases in DA may support well-being by enhancing motivation, activity, and exploratory behaviour [39,40], reducing fear responses [41], and promoting reproductive function [42] in animals. There is a need for further investigation into how different auditory stimulation environments may modulate neurodevelopmental pathways and behavioural outcomes in ducks and other avian species.

There is growing interest in the positive effects of PAS on poultry physiology. Research suggests that exposure to specific sounds during embryonic development can influence behaviour and stress responses in animals. For instance, embryos exposed to rhythmic sounds have been shown to develop more advanced nervous systems and exhibit reduced fear responses after hatching [43]. More recently, Ahmad-Hanafi et al [4] reported that prenatal exposure to noise and music improved the ability of broilers to cope with feed deprivation at 42 days of age, highlighting the potential of sensory stimulation in promoting resilience and well-being in poultry. According to Hedlund and Jensen [44], the process of incubation and hatching is potentially physiologically stressful. Interestingly, both MUSIC and NOISE ducklings exhibited lower HSP70 compared to the control group at PH1, suggesting that these groups were better equipped to cope with the physiological stresses associated with incubation and hatching [44]. Modulating HSP70 expression during embryonic development may enhance hatchability and survival rates, which is critical for commercial duck production and could improve resilience and adaptability to environmental challenges [45]. The benefits of PAS in enhancing stress resilience in hatchlings were also supported by the reduced CPN and OVT concentrations in the MUSIC and NOISE ducklings compared to their CONTROL counterparts at PH1. Hence, the present findings suggest that PAS can dampen physiological stress response following hatching in ducklings.

The underlying mechanism of PAS’s long-term impact on stress resilience is unknown. Stress early in life can induce long-term beneficial effects in poultry through epigenetic changes [46]. Epigenetic changes are genetic modifications that impact gene activity without changing the DNA sequence [47]. Antonson et al [48] showed that different acoustic playbacks *in ovo* alter genome-wide methylation of the auditory forebrain in late-stage zebra finch embryos. The applications of epigenetics to improve livestock production and welfare [49] merit further studies.

## CONCLUSION

PAS, as indicated by HSP70, induces a physiological stress response in developing embryos; however, it does not compromise hatchability rates or affect the weights of embryos and hatchlings. Our findings demonstrate that exposure to MUSIC and NOISE effectively alleviates the stress associated with incubation and hatching in day-old ducklings. This observation is notable and should be considered in future studies exploring early-life experiences and epigenetic modifications in avian species. The enhanced stress resilience observed in MUSIC- and NOISE-exposed ducklings at hatch has practical implications for developing strategies to improve the well-being of ducks within modern production systems, promoting both animal welfare and productivity.

## Data Availability

Upon reasonable request, the datasets of this study can be available from the corresponding author.
